# Robust data-driven incorporation of prior knowledge into the inference of dynamic regulatory networks

**DOI:** 10.1093/bioinformatics/btt099

**Published:** 2013-03-21

**Authors:** Alex Greenfield, Christoph Hafemeister, Richard Bonneau

**Affiliations:** ^1^Computational Biology Program, New York University Sackler School of Medicine, New York, NY 10065, ^2^Department of Biology, Center for Genomics and Systems Biology, New York, NY 10003 and ^3^Computer Science Department, Courant Institute of Mathematical Sciences, New York University, New York, NY 10012, USA

## Abstract

**Motivation:** Inferring global regulatory networks (GRNs) from genome-wide data is a computational challenge central to the field of systems biology. Although the primary data currently used to infer GRNs consist of gene expression and proteomics measurements, there is a growing abundance of alternate data types that can reveal regulatory interactions, e.g. ChIP-Chip, literature-derived interactions, protein–protein interactions. GRN inference requires the development of integrative methods capable of using these alternate data as priors on the GRN structure. Each source of structure priors has its unique biases and inherent potential errors; thus, GRN methods using these data must be robust to noisy inputs.

**Results:** We developed two methods for incorporating structure priors into GRN inference. Both methods [Modified Elastic Net (MEN) and Bayesian Best Subset Regression (BBSR)] extend the previously described Inferelator framework, enabling the use of prior information. We test our methods on one synthetic and two bacterial datasets, and show that both MEN and BBSR infer accurate GRNs even when the structure prior used has significant amounts of error (>90% erroneous interactions). We find that BBSR outperforms MEN at inferring GRNs from expression data and noisy structure priors.

**Availability and implementation:** Code, datasets and networks presented in this article are available at http://bonneaulab.bio.nyu.edu/software.html.

**Contact:** bonneau@nyu.edu

**Supplementary information:** Supplementary data are available at *Bioinformatics* online.

## 1 INTRODUCTION

Understanding how global regulatory networks (GRNs) coordinate systems-level response of a cell or organism to a new environmental state or perturbation is a key problem in systems biology, with applications spanning biofuels (Bonneau *et al.*, 2007), novel therapeutic targets (Carro *et al.*, 2010) and the discovery of novel pathways involved in cellular differentiation (Ciofani *et al.*, 2012). The cellular response is governed by multiple regulatory mechanisms that can be encapsulated by large network models. Recent advances in the quality and availability of high-throughput technologies enable measurement of different components of the GRN including mRNA transcript levels, protein levels, post-translational modifications, as well as DNA characteristics such as transcription factor-binding regions and open chromatin locations (ENCODE Project Consortium, 2012). These multi-level and multi-scale datasets have made the inference of integrative GRNs possible.

As high-throughput data capturing the abundance of mRNA transcripts are the most mature and readily available, many methods focus only on this single level of regulation, learning transcriptional regulatory networks. In transcriptional GRNs, the regulators are transcription factors (TFs, either previously known or predicted), and the targets are genes. Time-series data, capturing the temporal changes in transcript abundance, allow for the inference of the strength and direction of regulatory interactions, which can be used to predict how the system will behave under previously unmeasured conditions (Bonneau *et al.*, 2007). Here, we are primarily interested in methods for learning regulatory networks from compendia of expression data, and combining this data with complementary data sources that provide priors on network structure. Importantly, the priors we use in this work provide information about connectivity but do not provide any information about the relative strength, importance or dynamic properties of each known regulatory edge (these we atempt to learn from the data).

Learning networks from single data types has severe limitations, as GRNs operate on multiple levels in addition to the transcriptome; thus, alternate data types are needed to form a complete picture of cellular circuits. Even if one is interested in learning the purely transcriptional layer of a cell’s regulatory network, many TFs are post-transcriptionally modified in ways that confound single data-type network inference (the transcript abundance of a TF is not necessarily correlated with its protein abundance nor activity), and some regulatory sub circuits produce transcriptional output that is consistent with multiple models.

One way to mitigate these pitfalls is to use publicly available sources of complementary data with bearing on regulation. We term any data that contains direct TF-target information (either predicted, or experimentally validated) as structure priors. One source of such prior information is an ever-growing collection of experimentally validated and manually curated databases of regulatory interactions. These databases are especially rich for model organisms (Florez *et al.*, 2009; Gallo *et al.*, 2010; Gama-Castro *et al.*, 2011; Lammers *et al.*, 2010), and the sets of known regulatory interactions are considered to be accurate and precise (though not complete). Additionally, the ENCODE Project Consortium (2012) (a high-profile effort to build an encyclopedia of coding DNA elements) has generated a wealth of DNA-binding information that can be used to generate priors on mammalian regulatory network structure. These are only a few examples of an ever growing number of sources of GRN structure priors, and it can be seen that they differ substantially from organism to organism.

Each source of prior information on GRN structure is an incomplete recapitulation of the underlying network, and may contain many incorrect or irrelevant interactions. Thus, incorporating structure priors into expression-based GRN inference poses several interesting algorithmic challenges. Successful methods for integrative GRN inference must possess the following key properties: (i) The method must only include the part of the prior with support from the data. This is important, as the prior information typically is a collection of possible regulatory interactions, of which only a subset might be relevant in a given dataset. Also, this implies robustness to erroneous interactions in the prior, which can have various sources, such as non-functional TF binding reported by ChIP-Seq. (ii) Using a structure prior must not limit the ability to learn the part of the network for which no prior information exists. (iii) The user must be able to control the weight given to the prior. This feature allows the user to tune the method based on the believed completeness and accuracy of the prior, while respecting the first two properties over a wide range of parameters. In this work, we introduce two methods for incorporating structure priors that possess all three criteria.

### 1.1 Prior work

A lot of effort has been put into learning GRNs from gene expression time-series data and prior knowledge. For recent reviews on the topic we refer to Bar-Joseph *et al.* (2012) and Hecker *et al.* (2009).

Some of the first GRN inference methods allowed for the inclusion of additional data as structure priors (Imoto *et al.*, 2003; Tamada *et al.*, 2003). However, they allowed only for a very limited number of nodes in the network. Werhli and Husmeier (2007) and Husmeier and Werhli (2007) build on that work and express the available prior knowledge in terms of an energy function, from which a prior distribution over network structures is obtained. The complexity of these methods limits their application beyond small networks. More computational convenient methods use the static representation of known regulatory interactions to derive condition-specific topological changes in network structure (Ernst *et al.*, 2008; Luscombe *et al.*, 2004; Schulz *et al.*, 2012). Other methods combine expression data with prior knowledge to estimate transcription factor activities, which then allow to draw conclusions about the underlying network structure (Fu *et al.*, 2011; Seok *et al.*, 2009). Another method, similar to MEN proposed in this work, uses a network-constrained regularization procedure for linear models to incorporate prior information (Li and Li, 2008). However, in all these cases, it is not clear how sensitive these methods are to errors in the priors.

The need for benchmarking general methods for GRN inference using similar datasets and gold standard sets led to the organization of a field-wide test, the Dialogue for Reverse Engineering Assessments and Methods (DREAM) (Marbach *et al.*, 2012; Prill *et al.*, 2010; Stolovitzky *et al.*, 2007, 2009). The competitions have shown that methods that incorporated multiple data types in a mutually reinforcing manner typically performed better. However, in DREAM, all information about the networks, including gene names, were obfuscated from the participants; thus, methods that use any sort of prior information could not be tested.

### 1.2 Our approach

We extend the recently published Inferelator approach for GRN inference (Bonneau *et al.*, 2006, 2007; Greenfield *et al.*, 2010; Madar *et al.*, 2010) to incorporate structure priors into the inference procedure. We retain the core Inferelator ordinary differential equation model and introduce two separate model selection approaches that can use structure priors. One involves a modification of the Elastic-Net model selection approach, and we refer to it as Modified Elastic Net (MEN). This method has been introduced previously (Yong-a poi *et al.*, 2008; Zou and Zhang, 2009), and here we incorporate it into the Inferelator, and rigorously evaluate its performance. Additionally, we developed a novel model selection approach, which uses a Bayesian regression framework with Zellner’s g prior (Zellner, 1983) along with best subset selection for model selection. We refer to this method as Bayesian Best Subset Regression (BBSR).

We test MEN’s and BBSR’s ability to incorporate structure priors on three datasets: (i) the DREAM4 one-hundred node *in silico* challenge, (ii) the DREAM5 *Escherichia coli* dataset and (iii) a recently published *Bacillus subtilis* dataset (Nicolas *et al.*, 2012) (see Supplementary Material for additional description). As suggested by the DREAM consortium (Marbach *et al.*, 2012; Prill *et al.*, 2010; Stolovitzky *et al.*, 2007, 2009), we use area under the precision recall curve (AUPR) as the measure of performance. Importantly, for the *E.coli* and *B.subtilis* datasets, we evaluate performance only over the subset of genes and TFs that have at least one known interaction. We test the robustness of each method by supplying sets of structure priors that have incorrect prior information added. In doing so, we simulate the biologically relevant environment where literature- and experiment-derived priors will have incorrect and irrelevant information.

## 2 METHODS

We will first define our core model, a simple ordinary differential equation (ODE) model where transcription factors effect transcription rate, and where mRNA degradation rate is proportional to mRNA level. Following the description of our core model, we introduce two extensions, MEN and BBSR, to our prior method, the Inferelator, that enable the use of known regulatory edges to influence model selection.

### 2.1 Problem set-up

**Fig. 1.**
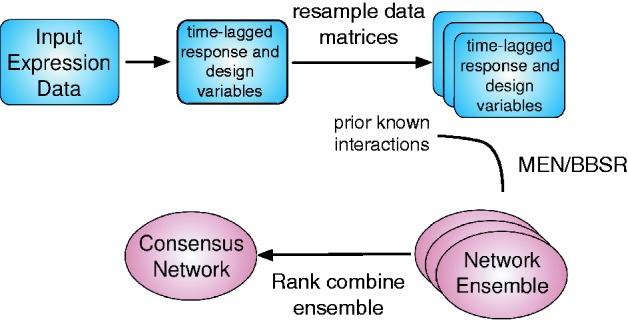
Method flow chart. Our method takes as input an expression dataset. To build a mechanistic model of gene expression, we create time-lagged response and design variables, such that the expression of the TF is time-lagged with respect to the expression of the target. We then resample the response and designing matrices, running model selection (using either MEN or BBSR) for each resample. This generates an ensemble of networks, which we rank combine into one final network

We define 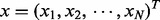 to be the observed mRNA expression levels of *N* genes, as measured by microarray (or RNAseq). The datasets contain two distinct sets of experiments: (i) time-series (*X^ts^*), and (ii) steady-state (*X^ss^*). In a time-series experiment, mRNA expression is measured at consecutive time points after some stimulus. To simplify notation, and without loss of generality, we assume that *X^ts^* is one such time series experiment, with *K* observations, 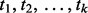 [i.e. 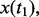 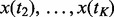 are the columns of *X^ts^*]. In a steady-state experiment, the mRNA expression is observed once, when the system has reached steady state. We consider all steady state experiments as *X^ss^* with *L* observations, 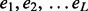 [i.e. 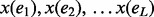 are the columns of *X^ss^*]. The method takes as input *X^ts^* and *X^ss^* and the output is a matrix *S*, where each entry 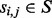 corresponds to the confidence that there exists a regulatory interaction between gene *x_j_* and gene *x_i_* (i.e. 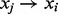). *S* can be thought of as a ranking of every possible regulatory interaction, where a higher value of 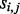 indicates a stronger confidence in 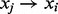. A flowchart summarizing our approach is depicted in Figure 1.

### 2.2 Limiting the number of regulators for each gene

When we infer transcriptional regulatory networks, we consider only *a-priori* known (or predicted) transcription factors as potential regulators. We define *P* to be the set of indices of the regulators in *x*. For each gene *i*, we have a specific set of regulators 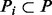. The members of *P_i_* are determined using tlCLR as in (Greenfield *et al.*, 2010; Madar *et al.*, 2010), and limited to the union of the 10 highest-scoring predictors and all predictors with prior knowledge. Note that we do not attempt to infer self-regulation in either method presented here, i.e. 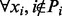.

### 2.3 Core model

We assume that the time evolution of the 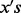 is governed by the following ODE
(1)
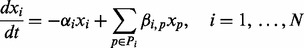

Where 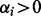 is the first order degradation rate [estimated from literature (Hambraeus *et al.*, 2003; Selinger *et al.*, 2003)], β is a set of parameters to be estimated and *P_i_* is the set of potential regulators for *x_i_*. For clarity, we describe the model formulation only for a linear combination of regulators, and note that as in Bonneau *et al.* (2006), this is easily extended to combinatorial interactions, and other non-linear functional forms. Recall that *x_i_* contains both time-series and steady-state observations, which we describe separately.

In the case of time-series data, we proceed by applying the finite difference approximation to the left hand side of Equation (1), isolating the unknown parameters β on the right hand side, and dividing both sides by 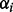. We can now write Equation (1) as
(2)
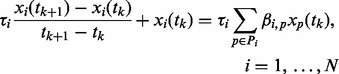

Where 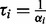 is related to the half-life 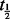 by 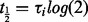. Note that here the design variable 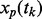 is time-lagged relative to the response variable 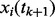 by one time point. This can easily be extended to consider a lag of multiple time points; however, multiple time-lags did not increase performance on the datasets tested here.

We summarize the left-hand side of the equation as *y_i_*, which we refer to as the time-series response variable, and approximate it as a linear combination of the *x_j_*’s, which we refer to as the time-series predictor (i.e. design variable). Over the time series conditions:
(3)
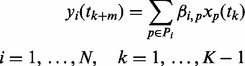



Note that the design and response variables are indexed only over the time-series conditions, and the design variables (*x_j_*’s) are time-lagged with respect to the response variable.

In the case of steady-state observations, 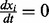, and Equation (1) becomes
(4)
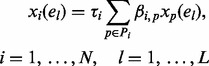



The two sides of the equation correspond to the steady-state response and design variables. To construct the final response and design variables, we concatenate the response and design variables over time-series and steady-state observations. The final step before model selection is to normalize and scale the response and design variables such that they have zero mean and variance of 1.

There are many ways to solve Equation (3), including regression. It was previously shown that sparse models of regulatory networks can accurately capture the topology and dynamics, and that using L1 shrinkage (and variations such as the Elastic-Net) can be used to enforce model parsimony (Greenfield *et al.*, 2010; Gustafsson and Hörnquist, 2010). Below, we describe MEN and BBSR, two different model selection procedures, both of which treat *y* as the response variables and the *x* as the predictor variables, learn parsimonious models, and have the ability to incorporate prior information.

### 2.4 Modified elastic net

*Algorithm Overview* Here we describe the MEN approach for estimating the parameters 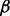 in Equation (3). We use MEN to both: (i) enforce a sparsity constraint on the parameters 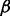, and (ii) incorporate prior knowledge of regulatory interactions 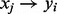. This approach has been previously described, but has never been rigorously tested in the context of incorporating constraints into GRN inference. We begin by describing the application of the Elastic-Net to model selection in the context of the core model described in Equation 3.

*Elastic-Net regression* The Elastic-Net (Zou and Hastie, 2005) finds a parsimonious solution to a regression problem [e.g. Equation (3)], and enforces sparsity through a penalty on the regression coefficients, which is a combination of the *l*_1_ lasso penalty, and the *l*_2_ ridge penalty. Let *R* be the total number of elements in response and design variable. We estimate the parameters 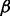 in Equation (3) by minimizing the following objective function (i.e. the sum of squares of the residuals).
(5)
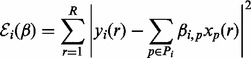

under the elastic net penalty on regression coefficients,
(6)
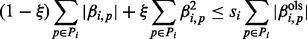

where 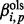 is the value of 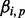 determined by ordinary least squares regression. ξ determines the balance between the lasso and ridge penalties, where 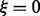 amounts to lasso regression, and 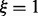 amounts to ridge regression. In practice, ξ is a vector, for each value of which we use 10-fold CV to pick *s_i_*. The final model for *y_i_* is determined by the value ξ and *s_i_*, which minimize the prediction error. This approach amounts to a grid search of the parameter space as described in Zou and Hastie (2005).

*Modified Elastic Net* To incorporate prior information directly into the model selection approach, we minimize Equation (5) subject to a new penalty function, closely related to Equation (6)
(7)
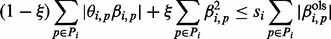

Where 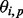 is a modifier on the shrinkage incurred on each parameter. If there is prior belief for a regulatory interaction 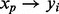, then 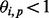 corresponds to less shrinkage being incurred on the corresponding 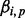, hence making it more likely that this parameter is not shrunk out of the model. Note that only the degree of shrinkage of a parameter is modified, not the correlation between a target, TF pair, nor the order in which predictors are selected by the model. In cases where multiple predictors are correlated (a common occurrence in biology), 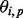 will cause predictors with no prior information to be shrunk from the model before predictors with prior information. Note that the 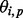 modifies only the *l*_1_ norm, as in Zou and Zhang (2009). This implementation is based on the elasticnet R package (Zou and Zhang, 2009).

### 2.5 Bayesian best subset regression

We now describe the BBSR method, an alternative inference method that computes all possible regression models for a given gene corresponding to the inclusion and exclusion of each predictor. Prior knowledge is incorporated by using informative priors for the regression parameters, and sparsity is enforced by a model selection step based on the Bayesian Information Criterion (BIC).

*Bayesian Regression With Informative Prior* Here we introduce the linear regression we use during the model building step of the algorithm. We assume the prediction error
(8)
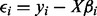

to be independent and identically distributed with mean 0 and variance 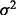. The response variable of gene *i* is denoted as *y_i_*, the design variables of TFs as *X* and the regression solution as 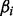. For clarity, we will omit the index *i* for the remainder of this section. We assume that the target gene response is distributed according to a multivariate normal
(9)
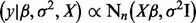

with the predicted response as mean, and a variance co-variance matrix that has the error variance 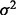 on its diagonal and is 0 otherwise. In this formulation, *n* is the number of observations (experiments). This can be solved by a Bayesian regression where we can incorporate existing knowledge by tuning the prior on β.

We use a modification of Zellner’s *g* Prior (Zellner, 1983) to include subjective information in our Bayesian regression problem. In the original formulation, the prior distribution of β has the following form
(10)
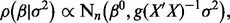

i.e. a distribution proportional to a multivariate normal with an initial guess 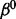 as mean and a data-dependent covariance matrix that is scaled by a user chosen factor of 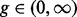. The prior distribution of 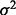 is the same as is typically used with the non-informative prior, 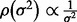. The choice of a large value for *g* will lead to results centred around the ordinary least squares solution, and the error variance will be the lowest. Values of *g* close to 0 on the other hand will lead to solutions that are centred around 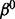 with higher error variance.

The joint posterior distribution has the functional form
(11)
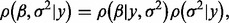

and the marginal posterior distributions are
(12)
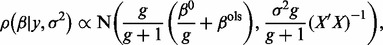

(13)
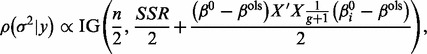

where 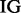 is the Inverse Gamma distribution with shape and scale parameter, and *SSR* is the sum of squares of the residuals of the ordinary least squares solution 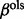.

With this set-up, we can propose a prior guess 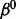 of the vector of regression coefficients, and encode our belief in this guess with *g*. To allow for different levels of confidence in the different elements of 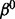, we extend the original formulation of the *g* prior to use a vector 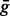 with one entry per predictor. The scale parameter of the Inverse Gamma distribution of the marginal posterior distribution of 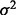 then becomes
(14)
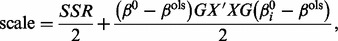

where *G* is a square diagonal matrix whose diagonal entries starting in the upper left corner are 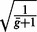 and all remaining entries are 0.

In practice, we choose 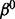 to be a vector with all entries having the value 0. This reflects our prior belief that the regulatory network is generally quite sparse. We set the vector 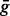 to values of *g* for those predictors that we have additional knowledge for and believe that they regulate gene *i*, and to 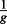 for the other predictors. A value of 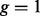 treats all predictors equally and we refer to it as ‘no priors’, whereas 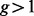 allows the predictors with priors to explain for more of the variance of the response.

*Model Selection* We use the BIC to select the final model from the 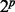 possible regression models for a gene *i*. For a given model *m*, the BIC is defined as
(15)
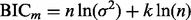

where *n* is the number of observations and *k* the number of predictors. To be more robust, we avoid using a point estimator for 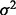 directly, but use the expected value of 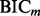 based on the posterior distribution of 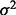

(16)
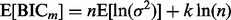

(17)
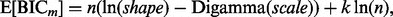

where *shape* and *scale* parameterize the marginal posterior distribution of 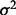 as in Equation 14. As a final step, the predictors of the model with the lowest 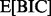 are selected as the TFs regulating gene *i*. If *p* is large (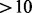), we use an initial filtration step to discover the 10 most promising predictors (see Supplementary Material for details).

### 2.6 Ranking interactions and bootstrapping

After model selection is carried out by either MEN or BBSR, the output is a matrix of dynamical parameters 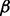, where each 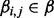 corresponds to the direction (i.e. activation or repression) and strength (i.e. magnitude) of a regulatory interaction. These parameters can be used to predict the response of the system to new perturbations. If the goal is to rank regulatory interactions based on a confidence measure, simply ranking by 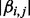 is not the best scheme, as this does not take into account the overall performance of the model for *y_i_*. As a result we re-rank interactions, taking into account the relative performance of each model, and the proportion of variance explained by each 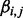. The result is a matrix *S* where the final confidence score for 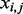 is given by
(18)
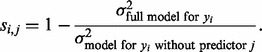



To further improve inference and become more robust against over-fitting and sampling errors, we use a bootstrapping strategy. We resample the input conditions with replacement and run model selection on the new dataset. This procedure is repeated 20 times, and the resulting lists of interactions (*S* matrices) are rank combined to a final ranked list as in Marbach *et al.* (2010).

## 3 RESULTS

We have conducted systematic thorough testing of the ability of both MEN and BBSR to accurately reconstruct GRNs using prior information in biologically relevant settings. We tested both methods with respect to the number and accuracy of prior known interactions (PKIs), and the effect of the weight of the PKIs. Performance is validated against the set of gold standard interactions (GSIs).

### 3.1 Effect of varying weight on priors

**Fig. 2.**
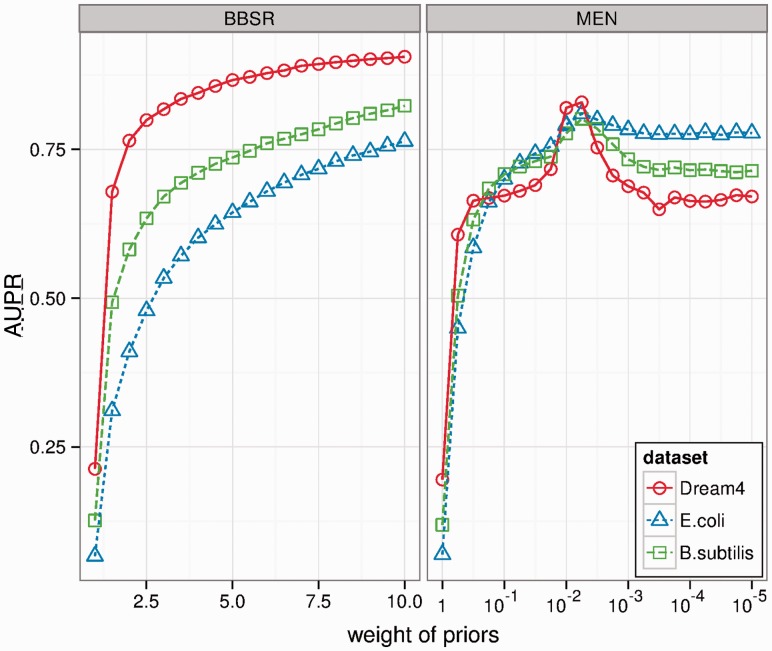
Effect of weight parameter on performance. We use all GSIs as the set of PKIs, and evaluate performance (in terms of AUPR) against the set of GSIs. We evaluate this performance for a variety of choices of the weight parameter for both methods

We assessed how sensitive our performance is to the choice of the weight parameter (θ for MEN and *g* for BBSR). For this initial investigation of parameter sensitivity, we used the entire gold standard as input (the set of PKIs covers all GSIs), and assessed performance over the set of GSIs. Though this design is circular, the purpose was to characterize the sensitivity of our method to the choice of θ and *g*, the parameters that control the relative influence of the structure prior for MEN and BBSR respectively (see Section 2). In Figure 2, we see the performance of each method (in terms of AUPR) as a function of the weight parameter. As the value of θ is decreased, the performance of MEN increases to a certain point, followed by a decrease in performance for all datasets (Fig. 2, right panel). This is true for all tested datasets, and it seems that MEN has a ‘sweet-spot’around 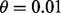, which results in best performance for all tested datasets. On the other hand, BBSR has a predictable behaviour for all tested datasets: performance increases for increasingly large values of *g*, limiting to an AUPR of 1 as *g* approaches infinity. This trend holds true for all datasets (left panel of Fig. 2).

### 3.2 Incorporation of prior interactions is data driven

We next investigated which of the known edges were included in the resulting network models. We used all GSIs as PKIs and selected a prior weight of 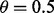 for all datasets for MEN, and values for *g* that resulted in similar AUPRs for BBSR (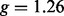, 2.2 and 1.6 for Dream4, *E.coli* and *B.subtilis*, respectively). We split the predicted interactions in two sets, high-ranked (recall 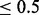) and low-ranked (recall 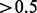 AND in set of PKIs), and compared the two sets with regard to the signal in the data. Signal for an interaction (TF-target pair) is defined as the time-lagged correlation for that pair. We chose this metric, as we use the time-lagged response and design matrices for model building (see Section 2).

**Fig. 3.**
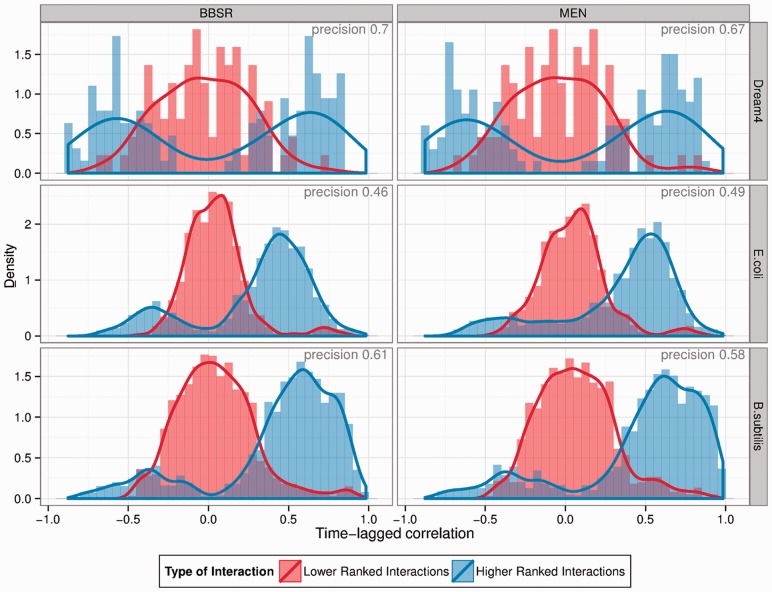
Incorporation of prior interactions is data driven. For all three datasets, we used all GSIs as PKIs. Here, we display the distribution of time-lagged correlation of predicted TF-target pairs at a recall level of 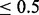 (higher ranked, blue), and low ranked interactions that are in the gold standard (lower ranked, red). Note that high ranked interactions are less likely to have low absolute time-lagged correlation, and the low ranked GSIs are centred around 0

For both methods and all datasets, we can see that high-ranked interactions have more signal (fewer near-zero correlations) than low-ranked interactions (densities peaked around zero), see Figure 3. However, for smaller values of θ, this trend is less pronounced for MEN, where more high-ranked interactions show time-lagged correlation of 0 (see Supplementary Material).

### 3.3 Performance on the leave-out set: using constraints does not damage our ability to learn new interactions

**Fig. 4.**
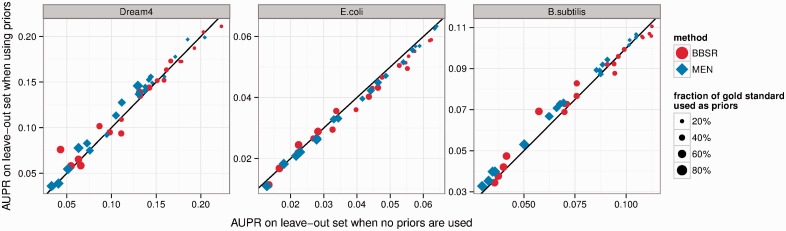
Performance change on the leave-out set. PKIs were sampled randomly from 20%, 40%, 60% and 80% of the GSIs in five repetitions. We define the leave-out set as the set of GSIs that are not PKIs. Here, we compare the AUPR of the leave-out set when using PKIs (*y*-axis) to the AUPR when not using PKIs (*x*-axis). Points above the line indicate a performance increase when PKIs are used

Here we assess if knowing part of the true regulatory network limits our ability to learn new regulatory interactions. We define the leave-out set as the set of GSIs that are not input as PKIs into our methods. For this experiment, we sampled PKI sets randomly resulting in subsets that consisted of 20, 40, 60 and 80% of the GSIs for each of the three datasets (we carried out five repetitions of this random sampling). We used the same weight parameters as in the previous section. AUPR of the leave-out set was computed when using PKIs and compared with the performance when no PKIs were used (Fig. 4). We observe similar trends for the six dataset–method combinations. Neither one method shows a consistent trend, and using prior information does not significantly help or damage performance on the leave-out set. However, very high weights for BBSR lead to a detectable performance decrease, whereas MEN is less affected by the prior weight (see Supplementary Material). Overall, performance on the leave-out set changes only slightly when priors are used.

In line with these observations, we can observe that overall performance increases linearly as the fraction of GSIs that is given as PKIs increases (see Supplementary Material). This trend is true for all three datasets and both methods.

### 3.4 Robustness to false prior information

**Fig. 5.**
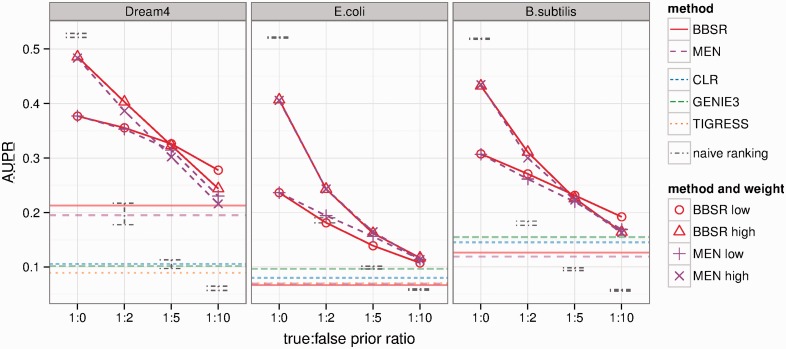
Robustness to incorrect prior information. For each dataset, we considered half of the GSIs as TPIs, and added varying numbers of FPIs that were not GSIs. We show the AUPR of both methods for multiple choices of the respective weight parameters, as well as methods that do not use any PKIs (horizontal lines). Additionally, we show the performance of a naive interaction ranking method, which places all PKIs at the top of the list (gray bars)

As sources of biological prior knowledge (e.g. literature-derived regulatory relationships, protein–protein interactions, ChIP-seq-detected binding events) are expected to have large numbers of incorrect (false prior) interactions, or interactions not relevant in a given dataset, it is important that methods for incorporating prior knowledge are robust to these cases. To test the robustness of MEN and BBSR to incorrect prior information, for each network, we considered half of the GSIs as true prior interactions (TPIs), and added a varying number of random false prior interactions (FPIs). We evaluated performance on the complete set of GSIs, and used as PKIs sets of interactions that have TPI:FPI ratios of 1:0, 1:2, 1:5, 1:10. A choice of 1:10 TPI:FPI for the *E.coli* dataset, for example, results in a set of PKIs that contains 1033 true interactions that are GSIs, and 10 330 false interactions which are not GSIs. FPIs were drawn randomly in five repetitions, and results showed a consistently low variance, so only mean values are presented here. We tested the performance of both MEN and BBSR on these PKI sets with increasing error for two choices of the respective weight parameters as follows. Low weights: θ for MEN is 0.5 for all datasets, and *g* for BBSR is 1.26, 2.2, 1.6 for Dream4, *E.coli*, *B.subtilis*. High weights: θ is 0.01 for all datasets, and *g* is 2.8, 13, 10. To compare our results with other methods, we used the web platform GenePattern (http://dream.broadinstitute.org/) and ran CLR, GENIE3 and TIGRESS on our data with default parameters. Additionally, we computed the AUPR of a simple interaction ranking method which places all PKIs at the top of the list. In general, high weight parameters make the methods more susceptible to noise, but for the two large datasets, *E.coli* and *B.subtilis*, performance throughout all noise levels is still better than any method without PKIs. For low weight parameters, and the Dream4 and *B.subtilis* datasets, BBSR is less susceptible to noise, and results in higher AUPRs than MEN (Fig. 5). For all three datasets, performance of both methods is always higher than the naive ranking scheme when false priors are present.

## 4 DISCUSSION

We developed two methods for incorporating prior knowledge into GRN inference. Both methods use the same underlying ODE model of regulation (see Section 2), but use different model selection approaches. MEN uses an adaptive weight on the penalty function to incorporate prior knowledge. BBSR uses the Bayesian formulation of linear regression, together with Zellner’s g-prior to incorporate prior information, and best subset regression with the BIC for sparse model selection.

A key difference between MEN and BBSR is how the choice of weight (how much influence to give to the prior) effects performance. Results presented in Figure 2 show that for BBSR higher values of *g* result in overall higher confidence in PKIs, and reduced confidence in all unknown interactions. As such, *g* can be interpreted as a confidence measure in the accuracy and completeness of PKIs, and be chosen accordingly. It is also possible to introduce multiple sources of prior information, each with a different weight (value of *g*). For MEN, the prior weight parameter θ exhibits a less predictable behaviour. Lower values of θ generally lead to higher confidence in PKIs. However, for all datasets, we observed a performance peak around 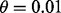. This non-linear property could be the result of cross-validation model selection procedure.

We tested the performance of both methods on different subsets of the GSIs. We see that increasing the number of PKIs increases performance in a linear manner for all datasets and both methods (Supplementary Fig. S2). This is in concordance with the results on the leave-out set (the set of GSIs that are not PKIs), where both methods showed only minor performance change in the presence of PKIs, regardless of dataset or number of PKIs used (Fig. 4).

Finally, and most importantly for application to biological systems where only incomplete and noisy sets of PKIs are available, we assessed the robustness of both methods to FPIs. Both methods are robust to FPIs, and outperform the naive ranking scheme, which assigns high confidence to all PKIs (Fig. 5). More specifically, for both large real datasets (*E.coli* and *B.subtilis*), both methods perform better than various baselines (no PKIs), with up to 10 FPIs for each true prior interaction. This means that both methods, given sufficient genomic data, are able to act as filters to distinguish between true and false prior interactions. However, BBSR is slightly more robust to the presence of FPIs.

A key consideration for any practical application of network inference methods with prior information is the trade-off between recapitulating the prior, and discovering novel biology. Intuitively, as the degree of belief in the prior is increased (by increasing the weight of the prior information), more of the interactions in the prior will be ranked highly by the inference method. Thus, high weights can lead to the incorporation of false interactions in the case of inaccurate PKIs (MEN more prone than BBSR), and impair performance on the leave-out set (as seen in BBSR). We suggest to the reader to set the weight parameter for incorporating prior knowledge based on the expected completeness and accuracy of the PKIs, and, when in doubt, to choose a low weight.

## 5 CONCLUSION

In this work, we have presented two methods for incorporating additional knowledge to constrain GRN inference by adding priors on the network structure. In the analysis of the methods, we focused on parameter choice and robustness to false priors, and show that both methods are remarkably tolerant to error in the priors. The inclusion of prior knowledge significantly improves the quality of inferred networks without damaging our ability to learn new interactions. Of our two methods, the BBSR inferred more accurate networks than the MEN in the presence of noise in the set of network priors used, and provides an intuitive weight parameter to control the strength of priors. This makes BBSR an appropriate method for integrating potentially noisy complementary data such as ChIP-Chip, ChIP-Seq, protein–protein interactions, literature-derived regulatory interactions and regulatory hypothesis derived from DNA-binding motifs into a data-driven regulatory network inference process.

## Supplementary Material

Supplementary Data
