# Supplementary material for: Robust data-driven incorporation of prior knowledge into the inference of dynamic regulatory networks
Source: Bioinformatics. 2013 Mar 21;29(8):1060–7. doi: 10.1093/bioinformatics/btt099 (PMC3624811; doi:10.1093/bioinformatics/btt099)
Supplement: Supplementary Data [file supp_29_8_1060__index.html]

Robust data-driven incorporation of prior knowledge into the inference of dynamic regulatory networks — Robust data-driven incorporation of prior knowledge into the inference of dynamic regulatory networks — Supplementary Data 

# Robust data-driven incorporation of prior knowledge into the inference of dynamic regulatory networks

## Supplementary Data

files

**Files in this Data Supplement:**

- Supplementary Data - pdf file
