# Supplementary material for: Robust data-driven incorporation of prior knowledge into the inference of dynamic regulatory networks
Source: Bioinformatics. 2013 Mar 21;29(8):1060–7. doi: 10.1093/bioinformatics/btt099 (PMC3624811; doi:10.1093/bioinformatics/btt099)
Supplement: Supplementary Data [file supp_btt099_priorsPaperSup.pdf]

Supplementary Material: Incorporating Prior Knowledge into Network Inference in a Robust, Data Driven Manner

Alex Greenfield *, Christoph Hafemeister *, and Richard Bonneau †

1 Reducing the number of predictors in Bayesian Best Subset Regression

For a given gene, the total number of potential predictors p in `BBSR` is determined by the size of the union of the 10 highest scoring predictors based on tlCLR and all those predictors that have previously been reported as regulators (see Method section in main article). If p is large (> 10) it becomes infeasible to compute all 2^p possible regression models during the model selection step. To further reduce the number of predictors, we look at a subset of of all possible models, and employ an averaging method to discover the 10 most promising predictors. We first build all models containing one or two predictors, and compute the expected BIC for each one. For every predictor we compute the average expected BIC of all models containing that particular predictor. These averages allow us to rank the predictors, and to reduce the set to the 10 best predictors as defined by the BIC in this small subspace of all models.

2 Datasets

For the performance tests of our methods described here, we use three datasets (Table 2).

1. A synthetic dataset from the DREAM4 [2] competition, consisting of 100 genes, 100 TFs (any gene can be a regulator), and 176 known (gold-standard) interactions. As this data is synthetic, normalization was taken care of by the organizers.

2. A real dataset characterizing the transcriptional changes in *E. coli*. The dataset comes from the DREAM5 challenge [5]. The majority of the data comes from the Many Microbes Microarrays Database (M3D), with a few

*These authors contributed equally to this work.
†To whom correspondence should be addressed.

additional arrays provided by the Collins lab [5]. Normalization was done using RMA [3]. The gold standard set of regulations contains 2066 interactions (characterizing 100 genes and 120 TFs) comes from RegulonDB followed by manual curation (removal of *in-silico* edges) manually by the organizers of DREAM. [5]

3. The final dataset we use for testing comes from the [6], characterizing the transcriptional dynamics of *B. subtilis*. This dataset consists of 269 tiling arrays capturing the response of *B. subtilis* to a variety of conditions. The gold standard comes from SubtiWiki [4, 1] which is repository of information for *B. subtilis*, including a manually curated list of known regulon information, which is what was used for the gold standard. Normalization and compilation of overlapping probes into intensities was done by the authors of the paper. We would also like to note to the reader that each of these datasets possess of markedly different amount of signal. We define signal as the time-lagged correlation between a TF and potential target (see Methods). We assessed the time-lagged correlation for all TF-target pairs, and only for those that were in the corresponding gold standard, and see the differences between the dataset in Fig. 1 below.

Dataset	Samples	Genes	TFs	Edges in gold standard
Dream4	421	100	100	176
E.coli	487	4511	334	2066
B.subtilis	269	4218	246	2422

Table 1: Dataset description

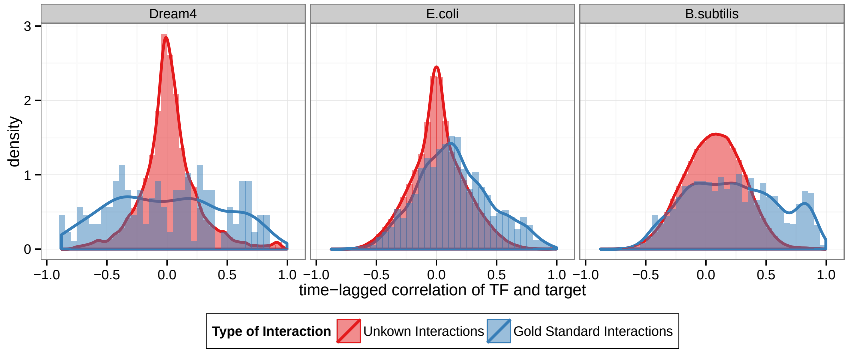

Figure 1: **Signal in dataset** Here we compute the signal present in each dataset. We consider the time-lagged correlation between every TF-target pair, and show the time-lagged correlations for interactions that are in the gold standard in blue, and in red we show the time-lagged correlations that are not in the gold standard.

3 Increasing the number of prior interactions predictably increases the number of gold standard edges included in model

Frequently in biological systems of interest, the set of PKIs is incomplete, covering only a small fraction of GSIs. To examine the relationship between the fraction of GSIs that were given as PKIs, and our performance, we increased the number of the PKIs for fixed weight parameters. The weight parameters were chosen as follows. High weights: θ for `MEN` is 0.01 for all datasets, and g for `BBSR` is 2.8, 13, 10 for Dream4, E.coli, B.subtilis. Low weights: θ is 0.5 for all datasets, and g is 1.26, 2.2, 1.6. We can observe that performance increases linearly as the fraction of GSIs that is given as PKIs increases, see Fig. 2. Although this analysis does not have bearing on our ability to predict new edges not in our gold standard it serves as an important verification that our methods for model selection do not exhibit strange behaviors as the number of known edges is either very small (as in the case for non-model organisms systems) or very large (as is the case for several model organisms, such as those used here, Yeast, Fly and Worm). In both cases optimal performance can be achieved in a relatively wide region of both θ and g suggesting that good values of these parameters can be estimated here based on these test data-sets and applied to a wide range of scenarios (differing amounts of noise, GS completeness, and data quantity).

This trend is true for all three datasets and both methods.

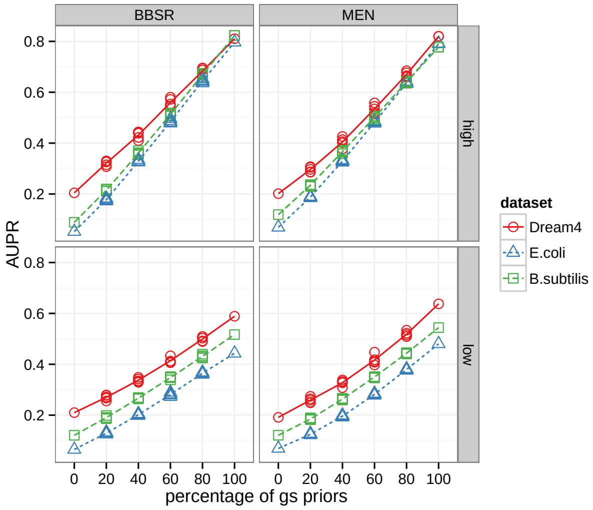

Figure 2: **Performance vs number of interactions in the prior** We supplied both methods with varying fractions of the gold standard interactions (GSIs) as prior known interactions, and evaluated performance (in terms of AUPR) over all GSIs. Fractions of GSIs were drawn at random in five repetitions. Here we show the results for high prior weights (top) and low prior weights (bottom).

3.1 Incorporation of prior interactions is data-driven

We investigated which of the known-edges were included in the resulting network models. We used all GSIs as PKIs and the weight parameters were chosen as follows. High weights: θ for `MEN` is 0.01 for all datasets, and g for `BBSR` is 2.8, 13, 10 for Dream4, E.coli, B.subtilis. Low weights: θ is 0.5 for all datasets, and g is 1.26, 2.2, 1.6. We split the predicted interactions in two sets, high-ranked (recall ≤ 0.5) and low-ranked (recall > 0.5 AND in set of PKIs), and compared the two sets with regard to the signal in the data. Signal for an interaction (TF-target pair) is defined as the time-lagged correlation for that pair. We chose this metric since we use the time-lagged response and design matrices for model building (see Methods section). For both methods and all datasets we can see that high-ranked interactions have more signal (fewer near-zero correlations) than low-ranked interactions (narrow densities peaked around zero), see Fig. 3. However, for high weights this trend is more pronounced for `BBSR`, where almost no high-ranked interactions show zero time-lagged correlation.

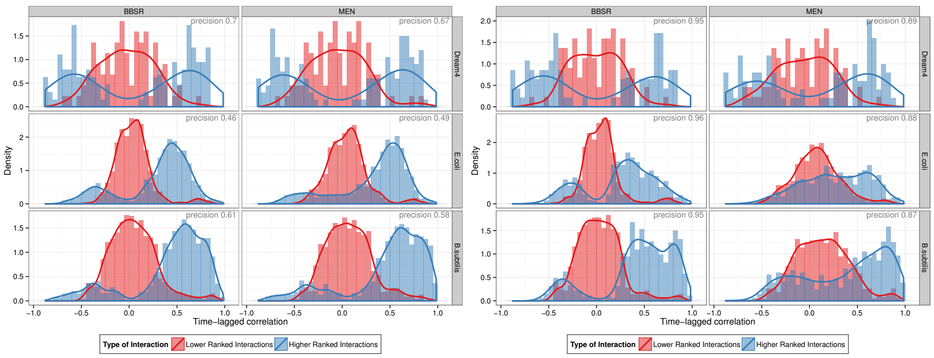

Figure 3: **Incorporation of prior interactions is data driven** For all three datasets we used all gold standard interactions (GSIs) as prior known interactions. Here we display the distribution of time-lagged correlation of predicted TF-target pairs at a recall level of ≤ 0.5 (higher ranked, blue), and low ranked interactions that are in the gold standard (lower ranked, red). Left panel: low weight parameters, right panel: high weight parameters. Note that high ranked interactions are less likely to have low absolute time-lagged correlation (less pronounced in `MEN` with high weight parameter), and the low ranked GSIs are centered around 0.

4 Performance on the leave-out set when using high weights

We define the leave-out set as the set of GSIs that are not input as PKIs into our methods. For this experiment we sampled PKI sets randomly resulting in subsets that consisted of 20%, 40%, 60%, and 80% of the GSIs for each of the three datasets (we carried out five repetitions of this random sampling). AUPR of the leave-out set was computed when using PKIs and compared to the performance when no PKIs were used, and the following weights were used: θ for `MEN` is 0.01 for all datasets, and g for `BBSR` is 2.8, 13, 10 for Dream4, E.coli, B.subtilis (Fig. 4). We observe that the performance on the leave-out set of `BBSR` degrades when high weights are used. This seems to be a small but consistent effect which cannot be seen for `MEN`. When compared to the results when using low weights (see main aricle), we can see that the performance of `MEN` on the leave-out set is independent of the choice of the weight parameter θ, whereas high values for g lead to a reduced performance on the leave-out set for `BBSR`.

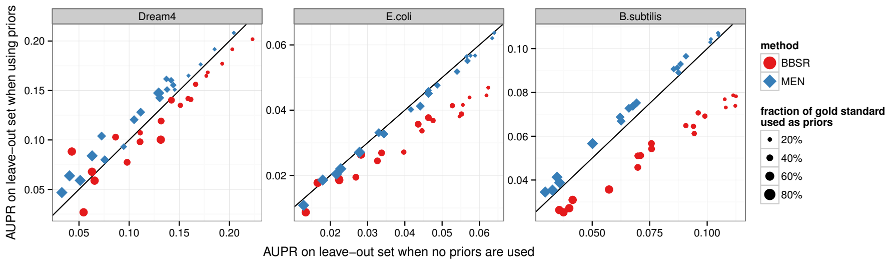

Figure 4: **Performance change on the leave-out set when using high weights.** Prior known interactions (PKIs) were sampled randomly from 20%, 40%, 60%, and 80% of the gold standard interactions (GSIs) in five repetitions. We define the leave-out set as the set of GSIs that are not PKIs. Here we compare the AUPR of the leave-out set when using PKIs (y-axis) to the AUPR when not using PKIs (x-axis). Points above the line indicate a performance increase when PKIs are used.

5 Running times

Dataset	MEN	BBSR
Dream4	01:02	00:35
E.coli	75:53	63:18
B.subtilis	60:14	44:04

Table 2: Averaged running times for one bootstrap in minutes CPU time (3GHz Intel Xeon).

References

[1] Lope A. Florez, Sebastian F. Roppel, Arne G. Schmeisky, Christoph R. Lammers, and Jorg Stulke. A community-curated consensual annotation that is continuously updated: the bacillus subtilis centred wiki subtiwiki. *Database*, 2009, 2009.

[2] Alex Greenfield, Aviv Madar, Harry Ostrer, and Richard Bonneau. Dream4: Combining genetic and dynamic information to identify biological networks and dynamical models. *PLoS ONE*, 5(10):e13397, 10 2010.

[3] Rafael A. Irizarry, Bridget Hobbs, Francois Collin, Yasmin D. BeazerBarclay, Kristen J. Antonellis, Uwe Scherf, and Terence P. Speed. Exploration, normalization, and summaries of high density oligonucleotide array probe level data. *Biostatistics*, 4(2):249–264, 2003.

[4] Christoph R. Lammers, Lope A. Florez, Arne G. Schmeisky, Sebastian F. Roppel, Ulrike Mader, Leendert Hamoen, and Jorg Stulke. Connecting parts with processes: Subtiwiki and subtipathways integrate gene and pathway annotation for bacillus subtilis. *Microbiology*, 156(3):849–859, 2010.

[5] Daniel Marbach, James C. Costello, Robert Küffner, Nicole M. Vega, Robert J. Prill, Diogo M. Camacho, Kyle R. Allison, The DREAM5 Consortium, Manolis Kellis, James J. Collins, and Gustavo Stolovitzky. Wisdom of crowds for robust gene network inference. *Nature methods*, 9(8):796–804, July 2012.

[6] Pierre Nicolas, Ulrike Mder, Etienne Dervyn, Tatiana Rochat, Aurlie Leduc, Nathalie Pigeonneau, Elena Bidnenko, Elodie Marchadier, Mark Hoebeke, Stphane Aymerich, Drte Becher, Paola Bisicchia, Eric Botella, Olivier Delumeau, Geoff Doherty, Emma L. Denham, Mark J. Fogg, Vincent Fromion, Anne Goelzer, Annette Hansen, Elisabeth Hrtig, Colin R. Harwood, Georg Homuth, Hanne Jarmer, Matthieu Jules, Edda Klipp, Ludovic Le Chat, Franois Lecointe, Peter Lewis, Wolfram Liebermeister, Anika March, Ruben A. T. Mars, Priyanka Nannapaneni, David Noone, Susanne Pohl, Bernd Rinn, Frank Rgheimer, Praveen K. Sappa, Franck Samson, Marc Schaffer, Benno Schwikowski, Leif Steil, Jrg Stlke, Thomas Wiegert, Kevin M. Devine, Anthony J. Wilkinson, Jan Maarten van Dijl, Michael Hecker, Uwe Vlker, Philippe Bessires, and Philippe Noirot. Condition-dependent transcriptome reveals high-level regulatory architecture in bacillus subtilis. *Science*, 335(6072):1103–1106, 2012.
